# Enhanced preoperative prediction of pancreatic fistula using radiomics and clinical features with SHAP visualization

**DOI:** 10.3389/fbioe.2025.1510642

**Published:** 2025-04-04

**Authors:** Yan Li, Kenzhen Zong, Yin Zhou, Yuan Sun, Yanyao Liu, Baoyong Zhou, Zhongjun Wu

**Affiliations:** ^1^ Department of Hepatobiliary Surgery, The First Affiliated Hospital of Chongqing Medical University, Chongqing, China; ^2^ Department of Radiology, The First Affiliated Hospital of Chongqing Medical University, Chongqing, China; ^3^ Department of Hepatobiliary Surgery, Bishan Hospital of Chongqing Medical University, Chongqing, China

**Keywords:** computed tomography, clinically relevant postoperative pancreatic fistula, machine learning, radiomics, the shapley additive explanations

## Abstract

**Background:**

Clinically relevant postoperative pancreatic fistula (CR-POPF) represents a significant complication after pancreaticoduodenectomy (PD). Therefore, the early prediction of CR-POPF is of paramount importance. Based on above, this study sought to develop a CR-POPF prediction model that amalgamates radiomics and clinical features to predict CR-POPF, utilizing Shapley Additive explanations (SHAP) for visualization.

**Methods:**

Extensive radiomics features were extracted from preoperative enhanced Computed Tomography (CT) images of patients scheduled for PD. Subsequently, feature selection was performed using Least Absolute Shrinkage and Selection Operator (Lasso) regression and random forest (RF) algorithm to select pertinent radiomics and clinical features. Last, 15 CR-POPF prediction models were developed using five distinct machine learning (ML) predictors, based on selected radiomics features, selected clinical features, and a combination of both. Model performance was compared using DeLong’s test for the area under the receiver operating characteristic curve (AUC) differences.

**Results:**

The CR-POPF prediction model based on the XGBoost predictor with the combination of the radiomics and clinical features selected by Lasso regression and RF exhibited superior performance among these 15 CR-POPF prediction models, achieving an accuracy of 0.85, an AUC of 0.93. DeLong’s test showed statistically significant differences (*P* < 0.05) when compared to the radiomics-only and clinical-only models, with recall of 0.63, precision of 0.65, and F1 score of 0.64.

**Conclusion:**

The proposed CR-POPF prediction model based on the XGBoost predictor with the combination of the radiomics and clinical features selected by Lasso regression and RF can effectively predicting the CR-POPF and may provide strong support for early clinical management of CR-POPF.

## 1 Introduction

Pancreaticoduodenectomy (PD) represents one of the most complex procedures within the surgical discipline and remains the gold standard for treating pancreatic and periampullary neoplasms (Bergeat et al., 2020). Despite significant advancements in surgical techniques and perioperative care, mortality rates in high-volume centers have been reduced to below 3% (Zureikat et al., 2016; Wu et al., 2023). However, CR-POPF persists as a major complication, occurring in 10%–20% of patients, leading to prolonged hospitalization, increased costs, and elevated morbidity and mortality (Ma et al., 2017; Williamsson et al., 2017; Hirono et al., 2019; Casciani et al., 2021). Early prediction of CR-POPF is critical for risk stratification and personalized management (Mungroop et al., 2019). Existing risk scoring models (Callery et al., 2013; Kantor et al., 2017; Mungroop et al., 2019; Mungroop et al., 2021), such as the Fistula Risk Score (FRS), rely on subjective intraoperative assessments (e.g., pancreatic texture) or postoperative parameters, limiting their utility for preoperative decision-making. Consequently, there is an urgent need for robust preoperative prediction tools that integrate objective, quantifiable biomarkers to guide clinical interventions.

Computed tomography (CT), widely used for preoperative evaluation, offers a non-invasive platform for objective risk stratification. However, conventional CT analysis focuses on macroscopic features (e.g., ductal morphology), which lack the granularity to capture subtle parenchymal heterogeneity linked to CR-POPF pathogenesis. Radiomics, an emerging paradigm, bridges this gap by converting medical images into high-dimensional quantitative features that reflect underlying pathophysiological processes (Gillies et al., 2016; Lambin et al., 2017; Rigiroli et al., 2021). These features, such as texture and shape parameters, quantify pancreatic fibrosis, microlobular fat infiltration, and ductal microcalcifications (Lubner et al., 2017; Chitalia and Kontos, 2019; Kim et al., 2019; Abunahel et al., 2022)—factors strongly associated with anastomotic integrity. Nevertheless, unimodal radiomics models often overlook systemic clinical variables (Huang et al., 2022; Tan et al., 2022; Mack et al., 2024), such as inflammatory markers or metabolic indices, which may synergize with imaging biomarkers to enhance predictive accuracy.

Machine learning (ML) provides a powerful framework to integrate radiomics with clinical data, enabling the development of multimodal predictive models. Prior studies demonstrate that combined models outperform unimodal approaches by capturing both microenvironmental heterogeneity and systemic physiological states (Capretti et al., 2022; Shen et al., 2022; Verma et al., 2024). For instance, texture features derived from gray-level matrices quantify pancreatic stiffness, while clinical variables like main pancreatic duct (MPD) diameter and platelet-to-albumin ratio (PAR) reflect anatomical risk and systemic inflammation, respectively. However, the clinical adoption of ML models has been hindered by their “black-box” nature, which obscures the interpretability of feature contributions (Azodi et al., 2020).

To address these challenges, we propose an interpretable ML framework that synergizes preoperative CT radiomics with clinical features for CR-POPF prediction. Our approach achieves superior predictive performance (AUC: 0.93) while addressing key limitations of existing methods—namely, their reliance on subjective intraoperative assessments, dependence on intraoperative or postoperative parameters, isolated use of unimodal data (radiomics or clinical features), and the opacity of traditional machine learning algorithms. By employing SHAP to elucidate feature contributions (Lundberg et al., 2020), we transform the model into a clinically interpretable tool. This integration not only enhances predictive accuracy but also provides mechanistic insights into how specific variables collectively influence fistula risk, bridging the gap between algorithmic performance and clinical trust.

## 2 Materials and methods

### 2.1 Study cohort

**FIGURE 1:**
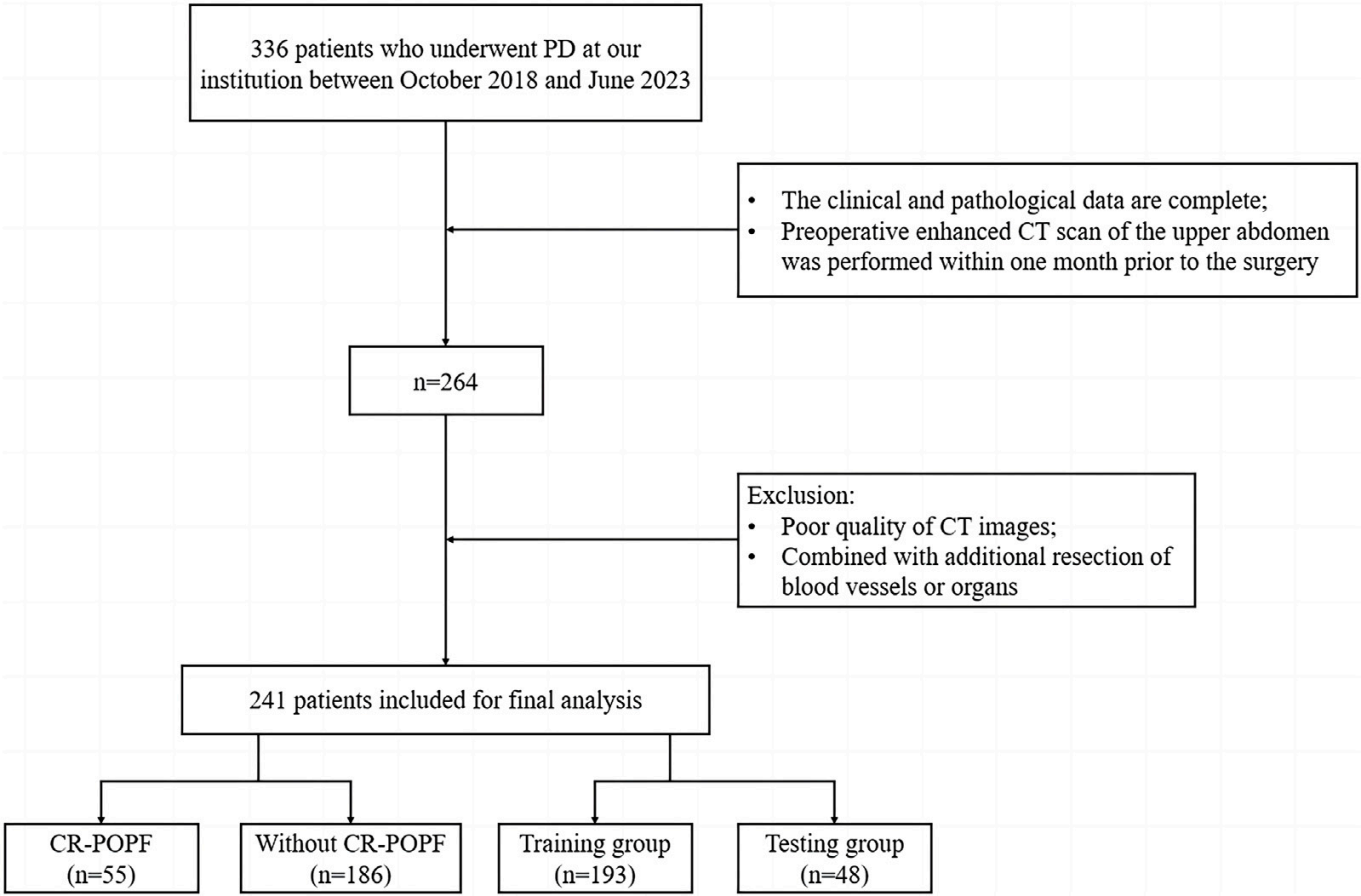
Flowchart of inclusion and exclusion criteria for eligible patients in the study. PD, pancreaticoduodenectomy; CT, computed tomography; CR-POPF, clinically relevant postoperative pancreatic fistula.

This retrospective cohort study was approved by the Ethics Committee of the First Affiliated Hospital of Chongqing Medical University (Ethics Approval Number: 2024-087-01). Informed consent was waived due to the retrospective design. We reviewed 336 patients who underwent PD between October 2018 and June 2023. Inclusion criteria were: (1) complete clinical and pathological data, (2) preoperative contrast-enhanced CT within 1 month before surgery. Exclusion criteria included: (1) non-curative resection, (2) prior neoadjuvant therapy, (3) poor CT image quality. After screening (Figure 1), 241 patients were included and stratified into CR-POPF (N = 55, 22.8%) and non-CR-POPF (N = 186, 77.2%) groups based on ISGPS 2016 criteria (Bassi et al., 2017).

Demographic and clinical comparisons between groups are summarized in Table 1. Age, gender, diabetes, hypertension, cardiovascular/pulmonary diseases, smoking history, and prior abdominal surgery showed no significant differences (*P* > 0.05). However, CR-POPF patients exhibited higher BMI (23.2 vs. 22.2 kg/m^2^, *P* = 0.035), increased alcohol consumption (43.6% vs. 28.0%, *P* = 0.042), and smaller MPD diameter (2.77 mm vs. 4.25 mm, *P* < 0.001). Preoperative laboratory tests revealed elevated platelet-to-albumin ratio (PAR: 6.55 vs. 5.53, *P* = 0.012) and bilirubin levels (129 vs. 78.2 μmol/L, *P* = 0.004) in the CR-POPF group. Pancreatic head lesions were more frequent in CR-POPF patients (67.3% vs. 32.7%, *P* = 0.006). No differences were observed in preoperative biliary drainage, ASA classification, or surgical approach (*P* > 0.05).

**TABLE 1:** Clinical baseline characteristics of patients.

Characteristic	Without CR-POPF	CR-POPF	P-value
	N = 186	N = 55	
Age (years)	61.4 (9.82)	60.7 (11.9)	0.685
Gender			0.186
Male	115 (61.8%)	40 (72.7%)	
Female	71 (38.2%)	15 (27.3%)	
BMI (kg/m^2^)	22.2 (3.20)	23.2 (2.86)	0.035
Diabetes mellitus	35 (18.8%)	6 (10.9%)	0.243
Hypertension	36 (19.4%)	14 (25.5%)	0.491
Heart disease	7 (3.76%)	5 (9.09%)	0.152
Lung disease	8 (4.30%)	4 (7.27%)	0.478
Drink	52 (28.0%)	24 (43.6%)	0.042
Smoke	72 (38.7%)	29 (52.7%)	0.090
Abdominal operation history	32 (17.2%)	11 (20.0%)	0.783
NLR	3.23 (2.34; 4.34)	3.45 (2.17; 4.97)	0.568
PLR	188 (141; 252)	181 (145; 272)	0.874
SII	688 (460; 1077)	820 (505; 1227)	0.136
PAR	5.53 (4.33; 7.26)	6.55 (5.27; 7.48)	0.012
Albumin (g/L)	39.0 (36.0; 42.8)	38.0 (35.0; 42.0)	0.509
Bilirubin (ummol/L)	78.2 (5.32; 149)	129 (33.7; 208)	0.004
ALT (U/L)	137 (41.5; 240)	95.0 (53.0; 197)	0.370
AST (U/L)	87.0 (33.5; 189)	82.0 (48.0; 136)	0.634
PBD	64 (34.4%)	26 (47.3%)	0.115
MPD (mm)	4.25 (3.06; 5.97)	2.77 (2.25; 3.94)	<0.001
Lesion location			0.006
Pancreas head	75 (40.3%)	37 (67.3%)	
Common bile duct	44 (23.7%)	8 (14.6%)	
Ampulla of Vater	42 (22.6%)	7 (12.7%)	
Duodenum	25 (13.4%)	3 (5.5%)	
ASA			0.196
I	0 (0.00%)	1 (1.82%)	
II	67 (36.0%)	17 (30.9%)	
III	116 (62.4%)	35 (63.6%)	
IV	3 (1.61%)	2 (3.64%)	
Approach			0.513
OPD	60 (32.3%)	21 (38.2%)	
LPD	126 (67.7%)	34 (61.8%)	

CR-POPF, clinically relevant postoperative pancreatic fistula; BMI, body mass index; NLR, neutrophil-to-lymphocyte ratio; PLR, platelet-to-lymphocyte ratio; SII, systemic immune-inflammation index; PAR, platelet-to-albumin ratio; ALT, alanine aminotransferase; AST, aspartate aminotransferase; PBD, preoperative biliary drainage; MPD, main pancreatic duct diameter; ASA, american society of anesthesiologists; OPD, open pancreaticoduodenectomy; LPD, laparoscopic pancreaticoduodenectomy.

The cohort was randomly split into training (n = 193, 80%) and test sets (n = 48, 20%) using an 8:2 ratio. Reporting followed TRIPOD guidelines (Collins et al., 2015).

### 2.2 CT technique

Contrast-enhanced abdominal CT scans were performed using Siemens SOMATOM Force, GE Discovery CT750 HD, or GE LightSpeed VCT. Scanning parameters: 120 kV, 200 mA, 5 mm slice thickness. All images were reconstructed using a standard reconstruction kernel with the following parameters: pitch of 1, rotation time of 0.5 s, field of view of 350 mm × 350 mm, matrix size of 512 × 512, slice thickness of 5 mm, interval of 5 mm, and reconstruction slice thickness of 1 mm. Patients were required to fast and avoid drinking for at least 3 h prior to the examination. A non-ionic iodinated contrast agent (300–400 mgI/ml) was administered intravenously at a dose of 1–1.5 mL/kg with an injection rate of 3 mL/s. Arterial phase scanning was delayed by 15–18 s. Portal venous and delayed phase scans were performed with delays of 33–36 s and 180 s, respectively. Enhanced CT images were exported from the Picture Archiving and Communication System (PACS) in DICOM format for further analysis.

### 2.3 Image preprocessing and segmentation

Image preprocessing included artifact removal, grayscale normalization (0–255), and enhancement via contrast adjustment, sharpening, and noise reduction.

Two radiologists (>5 years of experience) manually delineated pancreatic parenchyma (body and tail) as regions of interest (ROIs) on portal venous phase images using ITK-SNAP (v3.6.0). The portal vein served as the anatomical landmark to differentiate the pancreatic head from the body. Segmentation masks were saved in Nifti format. A senior radiologist (>10 years of experience) validated 50 randomly selected samples. Intraclass and interclass correlation coefficients (ICCs) were calculated, with ICC >0.8 indicating satisfactory reproducibility.

### 2.4 Feature extraction and selection

Radiomics feature extraction was performed using the PyRadiomics library (v3.0.1) in Python, based on original CT images and their preprocessed variants, including those filtered with Laplacian of Gaussian (LoG) and wavelet transforms. The extracted features encompassed first-order statistics (e.g., mean, variance, skewness), shape features (e.g., volume, sphericity, maximum diameter), and texture features derived from matrices such as the gray-level co-occurrence matrix (GLCM), gray-level run-length matrix (GLRLM), gray-level size zone matrix (GLSZM), and neighborhood gray-tone difference matrix (NGTDM).

To optimize feature selection and reduce dimensionality, the Lasso regression combined with cross-validation (Lasso-CV) was applied. Regularization parameters were optimized using grid search, and features with non-zero coefficients were retained. Additionally, the Random Forest (RF) algorithm was employed to rank the importance of clinical variables and identify the most relevant features for model development.

### 2.5 Model construction and validation

In the training cohort, five machine learning predictors—XGBoost, Random Forest (RF), Extra Trees (ET), Gradient Boosting (GB), and AdaBoost—were employed to develop 15 CR-POPF prediction models (the model parameters are presented in Table 2). These models were trained on three datasets: radiomics-only, clinical-only, and a combined radiomics-clinical dataset. Feature selection and model training were performed exclusively on the training set. The test set remained entirely independent and was only used for final model evaluation to prevent data leakage. Hyperparameters, including learning rate, maximum tree depth, subsampling rate, and regularization terms, were optimized via 5-fold cross-validated grid search to balance model complexity and generalizability. To mitigate overfitting, early stopping mechanisms and maximum iteration limits (10,000 iterations) were enforced during training. Model performance was rigorously evaluated using accuracy, AUC, precision, recall, and F1 score.

**TABLE 2:** Hyperparameters of machine learning models.

Model	Parameter	Value
XGBoost	n_estimators	100
	learning_rate	0.3
	max_depth	6
	subsample	1
	colsample_bytree	1
Random Forest	n_estimators	100
	max_depth	None
	min_samples_split	2
	min_samples_leaf	1
Extra Trees	n_estimators	100
	max_depth	None
	min_samples_split	2
	bootstrap	False
Gradient Boosting	n_estimators	100
	learning_rate	0.1
	max_depth	3
	subsample	1.0
AdaBoost	n_estimators	50
	learning_rate	1.0
	estimator	DecisionTreeClassifier (max_depth = 1)

All models were implemented using Python’s scikit-learn (v1.2.2) and XGBoost (v1.7.6) libraries. Unspecified parameters retained their default values.

**FIGURE 2:**
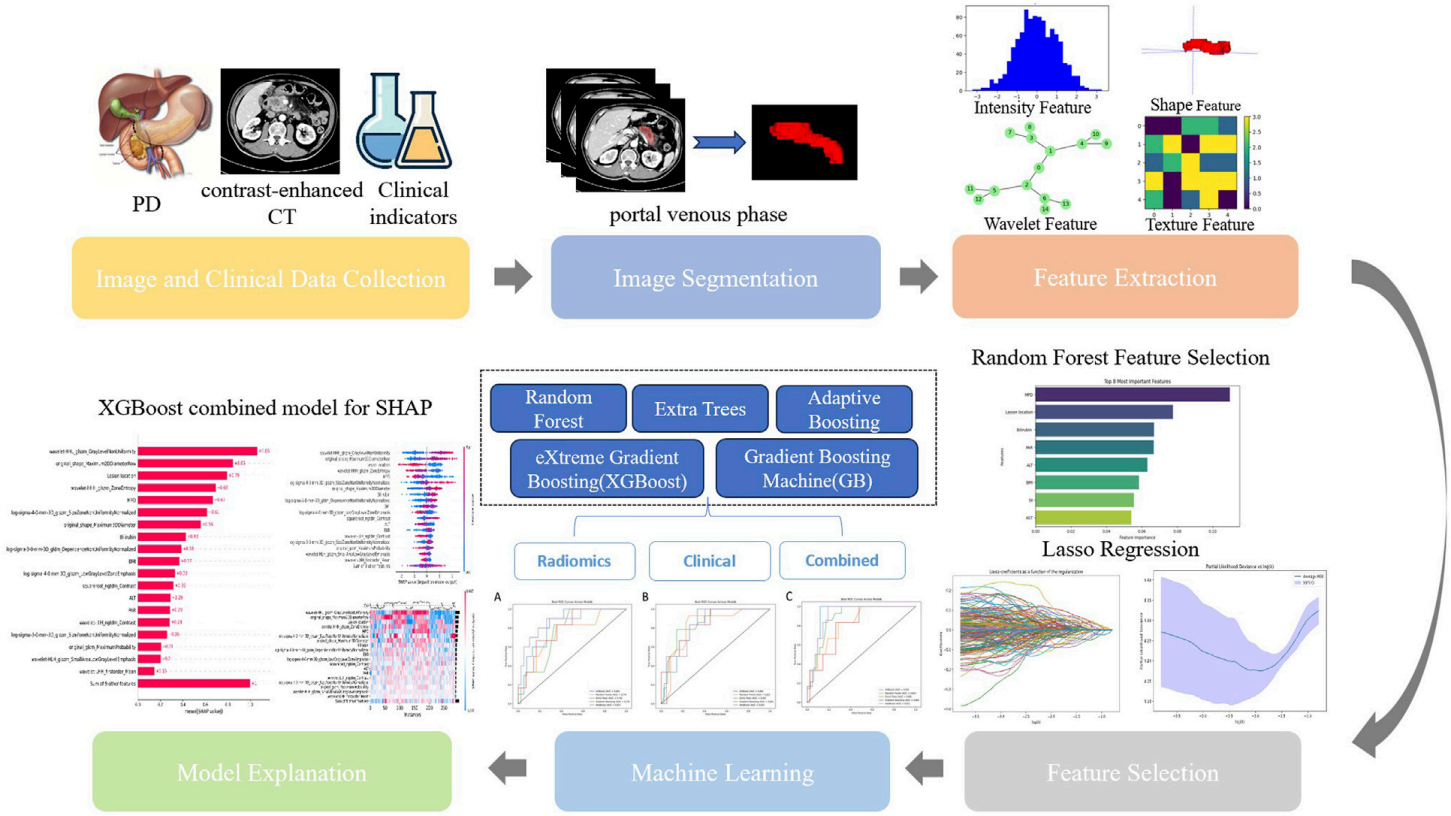
Workflow of model development. PD, pancreaticoduodenectomy; CT, computed tomography; Lasso, Lasso regression; SHAP, Shapley Additive explanation.

Pairwise comparisons of AUC values between models were conducted using DeLong’s test, with results visualized as a heatmap (Supplementary Material S1) to highlight statistically significant differences (P < 0.05). Calibration curves quantified the agreement between predicted probabilities and observed outcomes, while decision curve analysis (DCA) assessed clinical utility by quantifying net benefits across threshold probabilities (Supplementary Material S2). Model interpretability was enhanced via SHAP analysis, elucidating feature contributions globally and locally. The workflow is summarized in Figure 2.

### 2.6 Evaluation metrics

To comprehensively evaluate the performance of the predictive models, five standard metrics were employed: accuracy, precision, recall, F1-score, and AUC. The definitions and corresponding formulas are as follows:

• Accuracy: The proportion of correctly classified instances relative to the total instances (Sokolova and Lapalme, 2009).

$$\text{Accuracy}=\frac{TP+TN}{TP+TN+FP+FN}$$

• Precision: The proportion of true positive predictions among all positive predictions (Sokolova and Lapalme, 2009).

$$\text{Precision}=\frac{TP}{TP+FP}$$

• Recall: The proportion of true positive predictions among all actual positive instances (Sokolova and Lapalme, 2009).

$$Recall=\frac{TP}{TP+FN}$$

• F1-score: The harmonic mean of precision and recall (Sokolova and Lapalme, 2009).

$$F1-score=2\times \frac{Precision\times Recall}{Precision+Recall}$$

• AUC: The area under the receiver operating characteristic (ROC) curve, calculated as the probability that a randomly chosen positive instance is ranked higher than a negative instance. For *M* positive and *N* negative instances (Fawcett, 2006):

$$\text{AUC}=\frac{\sum_{i=1}^{M}\sum_{j=1}^{N}I\left(P_{i}>P_{j}\right)}{M\times N}$$

where *Pi* and *Pj* denote the predicted probabilities of the *i*-th positive and *j*-th negative instance, respectively, and *I* (⋅) is an indicator function equal to 1 if *Pi* > *Pj*.

### 2.7 statistical analysis

The statistical evaluations were executed employing Python software (version 3.7; https://www.python.org/). Quantitative data, conforming to a normal distribution, are articulated as the mean ± standard deviation (SD), while quantitative data that do not follow a normal distribution are represented as the median, along with the interquartile range. Categorical data are denoted as numbers and percentages (N, %). To assess the efficacy of the constructed models, several widely utilized metrics were chosen, encompassing accuracy, precision, recall, F1 score, and the area under the Receiver Operating Characteristic (ROC) curve (AUC). Pairwise comparisons of AUC values between models were conducted using DeLong’s test to assess statistical significance. The threshold for statistical significance was established at P < 0.05.

## 3 Results

### 3.1 Feature selection outcomes

**FIGURE 3:**
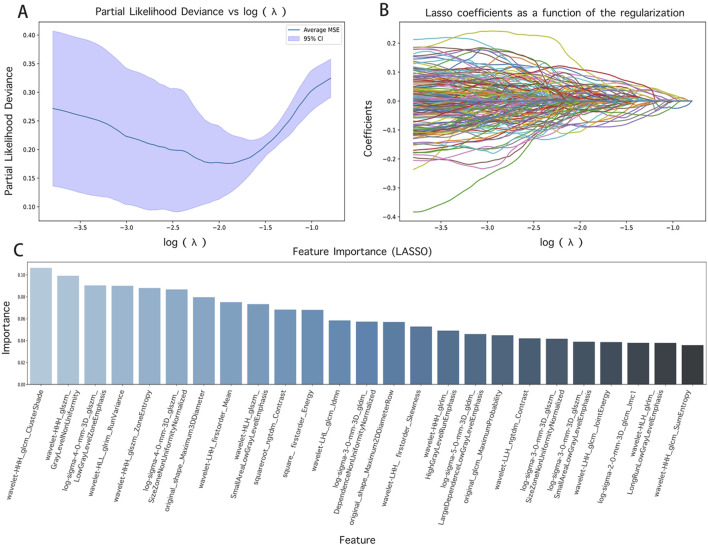
Feature screening results and their visualization. **(A)** and **(B)** are the results of different α parameters in the Lasso algorithm; **(C)** is the radiomics features with contribution values not equal to 0.

In the course of this study, the Pyradiomics library was utilized to derive 1719 radiomics features from CT images. To guarantee the performance and interpretability of the model, Lasso regression was implemented for the selection of these high-dimensional features. The alterations in the model’s performance with varying parameter α iterations were depicted in Figures 3A, B, thereby determining the optimal parameter values and the corresponding number of features. The Lasso regression model was then used to pinpoint features with non-zero coefficients, which were subsequently ranked based on the absolute values of these coefficients, as illustrated in Figure 3C, including texture features (e.g., wavelet HHL_glszm_GrayLevelNonUniformity, original_glcm_ClusterProminence) and shape features (e.g., original_shape_Maximum2DDiameterRow, original_shape_Sphericity).

**FIGURE 4:**
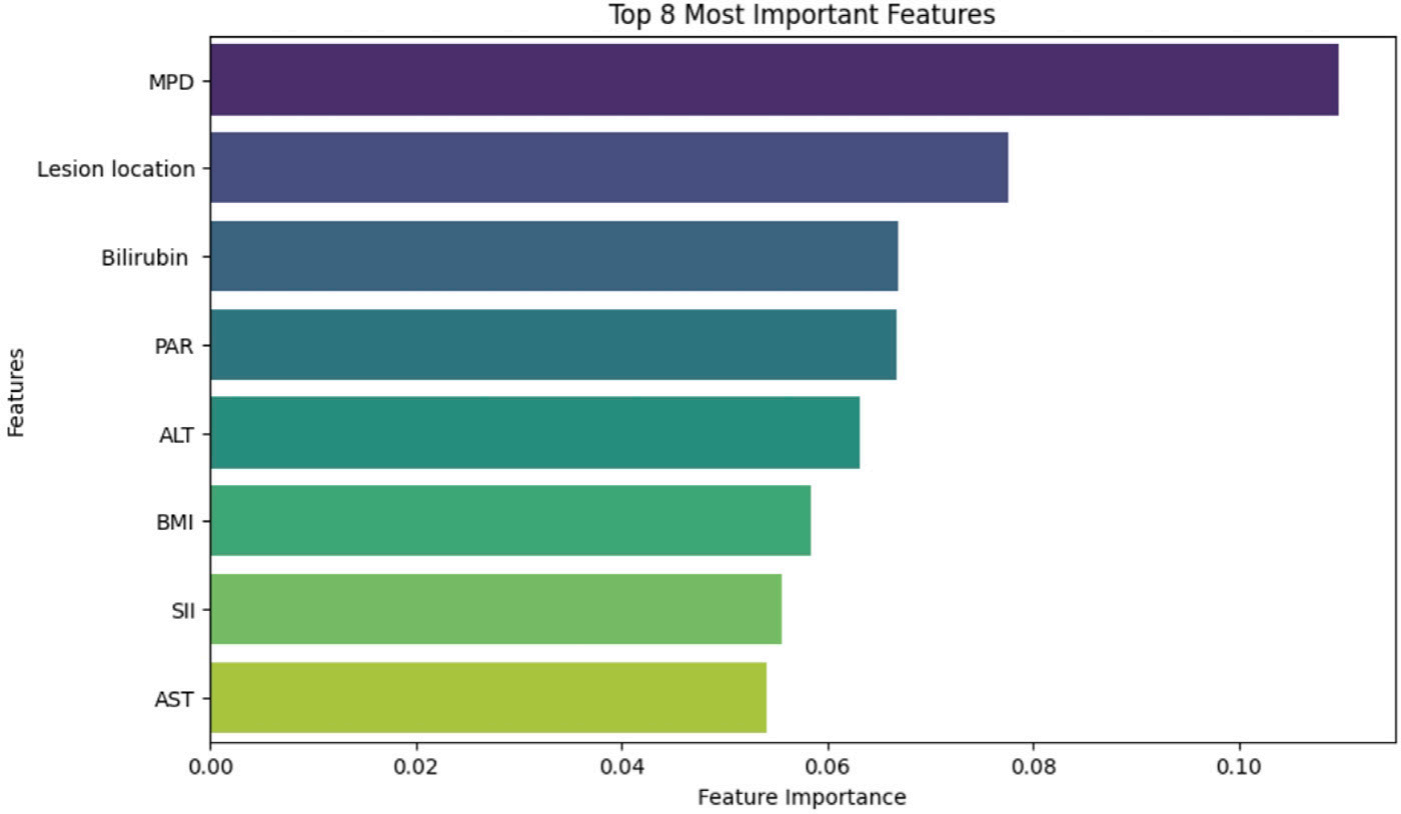
Contribution ranking of key clinical features selected using Random Forest analysis.

For clinical features, RF analysis ranked eight predictors by importance (Figure 4): MPD diameter (2.77 mm vs. 4.25 mm in CR-POPF vs non-CR-POPF), lesion location (67.3% vs. 32.7% pancreatic head involvement), bilirubin (129 vs. 78.2 μmol/L), PAR (6.55 vs. 5.53), ALT (95.0 vs. 137 U/L), BMI (mean: 23.2 vs. 22.2 kg/m^2^), systemic immune-inflammation index (SII) (820 vs. 688), and AST (82.0 vs. 87.0 U/L).Combining radiomics and clinical features, we established a multimodal feature set encompassing 28 variables for subsequent training and validation of ML models.

### 3.2 Model performance comparison

**FIGURE 5:**
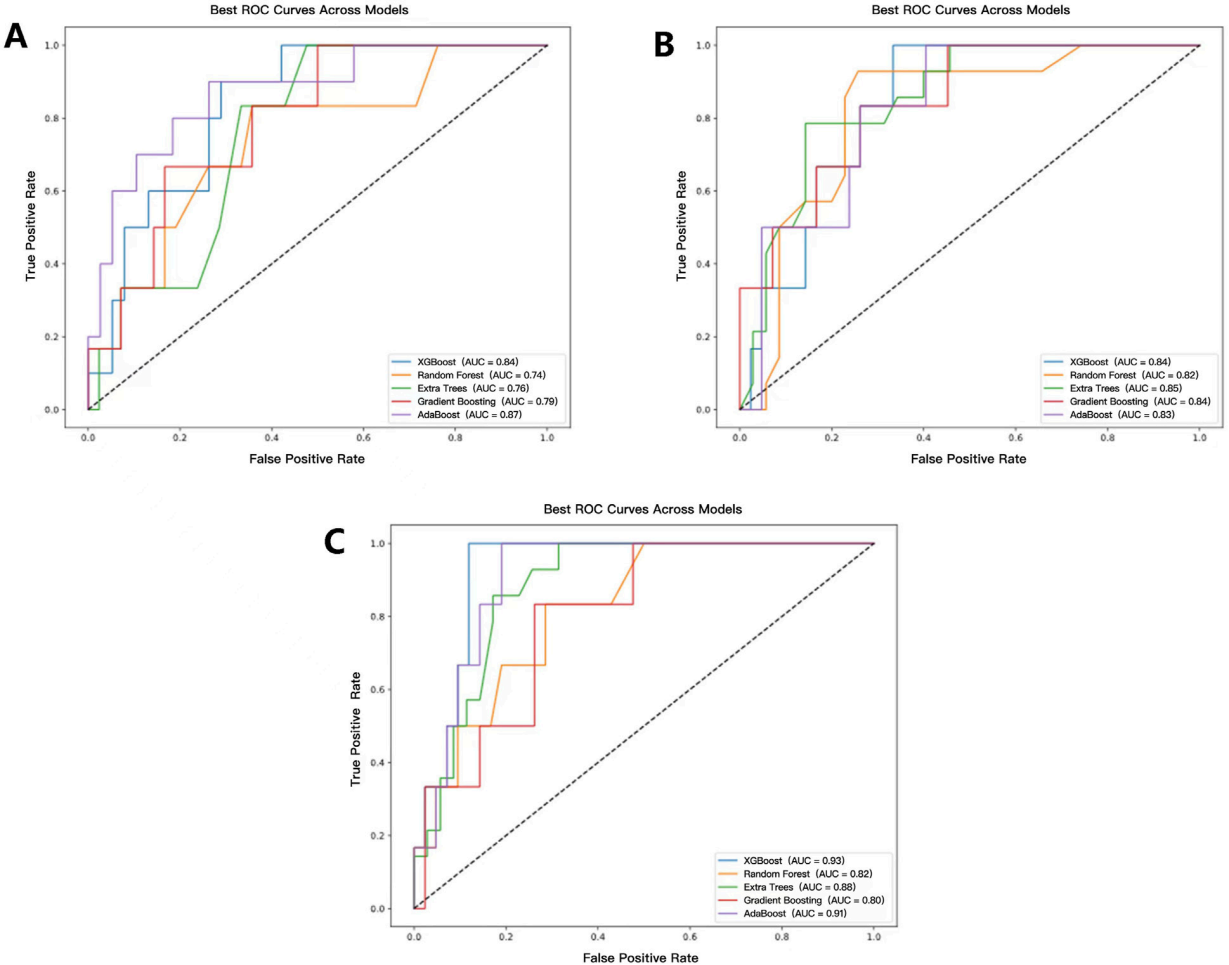
ROC curves and AUC values of each model. **(A–C)** represent the prediction model results based on radiomics, clinical, and radiomics-clinical, respectively.

A total of fifteen CR-POPF prediction models were developed using five distinct ML predictors, incorporating selected radiomics features, selected clinical features, and a combination of both. Among the radiomics-based models, the AdaBoost model demonstrated the highest predictive performance, achieving the highest AUC of 0.87 (Figure 5A), along with the best recall (0.76) and precision (0.71). In contrast, the RF and Extra Trees models exhibited the highest accuracy (0.83); however, the RF model showed lower robustness, with an AUC of 0.74. Regarding clinical models, the Extra Trees model achieved the highest AUC (0.85, Figure 5B) while maintaining a balanced performance in terms of precision (0.75) and recall (0.75).

The CR-POPF prediction model based on the XGBoost predictor with the combination of the selected radiomics and clinical features demonstrated superior performance among all 15 CR-POPF prediction models, achieving an accuracy of 0.85 and an AUC of 0.93 (Figure 5C). Compared to unimodal models, the combined model exhibited statistically significant improvements. Specifically, when compared to the radiomics-only model using the Extra Trees predictor, the AUC difference was 0.17 (*p* = 0.041), and when compared to the clinical-only model using the Random Forest predictor, the AUC difference was 0.11 (*p* = 0.041). These results, validated by DeLong’s test (Supplementary Material S1, where red cells denote *p* < 0.05), highlight the synergistic value of multimodal integration.

Detailed performance metrics are summarized in Table 3, while calibration and decision curve analysis (DCA) curves (Supplementary Material S2) further validated the clinical utility of the combined model across threshold probabilities. These results highlight that the integration of CT radiomics and clinical data significantly enhances preoperative CR-POPF risk stratification.

**TABLE 3:** Performance comparison of each model.

Feature	Model	Accuracy	Recall	Precision	F1 score
Radiomics	XGBoost	0.79 ± 0.06	0.68 ± 0.08	0.68 ± 0.10	0.68 ± 0.08
	RF	0.83 ± 0.08	0.55 ± 0.04	0.57 ± 0.06	0.55 ± 0.04
	Extra Trees	0.83 ± 0.08	0.55 ± 0.04	0.57 ± 0.07	0.55 ± 0.05
	GB	0.79 ± 0.07	0.67 ± 0.09	0.61 ± 0.11	0.63 ± 0.10
	AdaBoost	0.79 ± 0.06	0.76 ± 0.11	0.71 ± 0.09	0.72 ± 0.09
Clinical	XGBoost	0.77 ± 0.03	0.73 ± 0.06	0.63 ± 0.02	0.64 ± 0.05
	RF	0.78 ± 0.06	0.71 ± 0.08	0.72 ± 0.08	0.72 ± 0.08
	Extra Trees	0.80 ± 0.06	0.75 ± 0.09	0.75 ± 0.07	0.75 ± 0.08
	GB	0.81 ± 0.08	0.75 ± 0.09	0.65 ± 0.08	0.68 ± 0.08
	AdaBoost	0.73 ± 0.06	0.77 ± 0.11	0.63 ± 0.08	0.63 ± 0.09
Radiomics-Clinical	XGBoost	0.85 ± 0.05	0.63 ± 0.04	0.65 ± 0.06	0.64 ± 0.04
	RF	0.85 ± 0.07	0.70 ± 0.08	0.68 ± 0.09	0.69 ± 0.09
	Extra Trees	0.76 ± 0.04	0.61 ± 0.08	0.71 ± 0.09	0.62 ± 0.02
	GB	0.81 ± 0.04	0.68 ± 0.05	0.63 ± 0.04	0.64 ± 0.04
	AdaBoost	0.83 ± 0.07	0.76 ± 0.11	0.67 ± 0.09	0.70 ± 0.09

### 3.3 XGBoost combined model for SHAP

**FIGURE 6:**
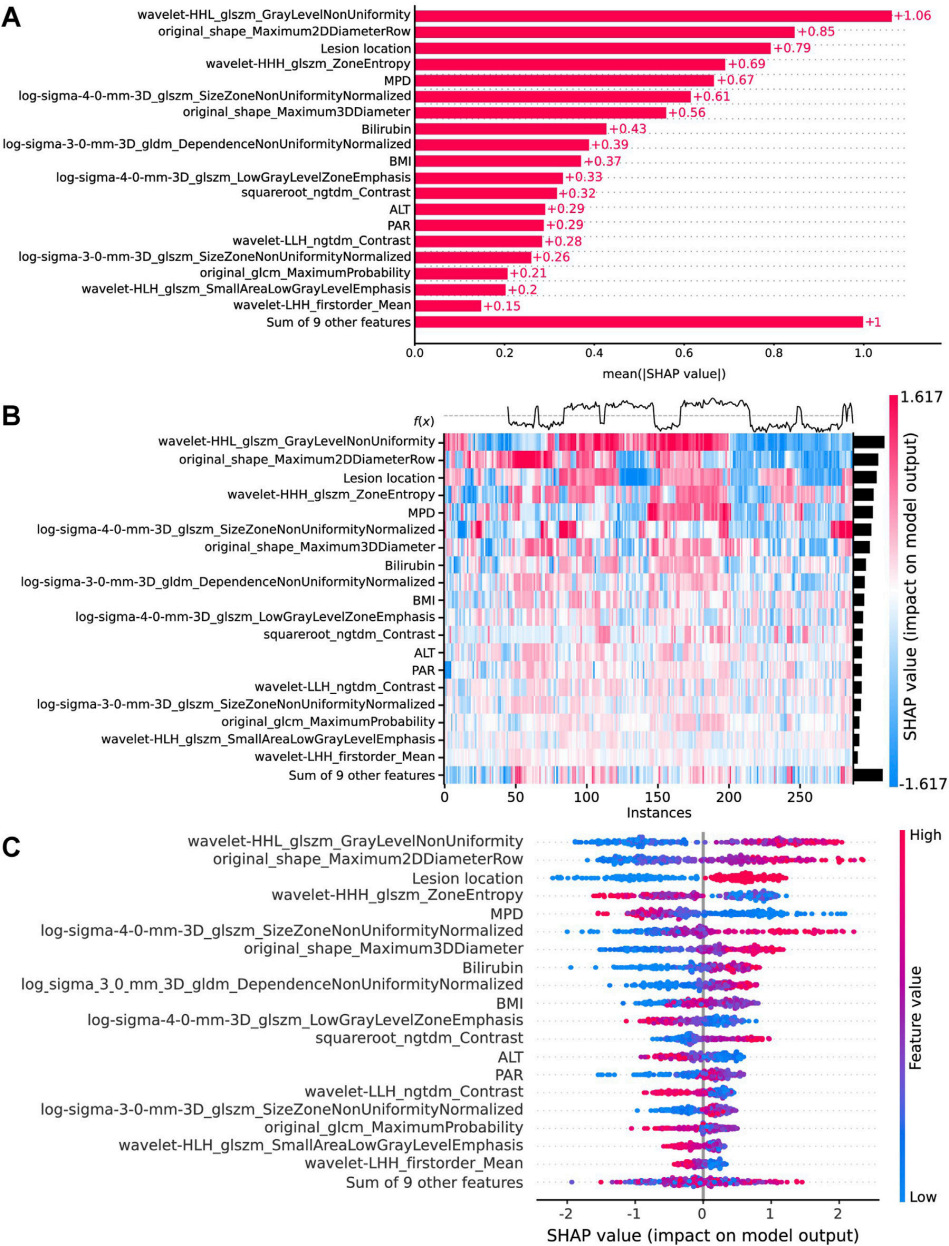
Global model explanation by the SHAP method. **(A)** The SHAP bar chart. **(B)** The SHAP heatmap plot shows the direction and intensity of influence for each feature of all cases in the model. **(C)** The SHAP beeswarm plot for the top 20 features in the model. Each dot represents a patient for each feature, with red denoting a higher feature value and blue denoting a lower feature value. The x-axis represents the SHAP values that describe the impact of each feature on model prediction. Positive SHAP values indicate an increased risk of CR-POPF, whereas negative SHAP values indicate a decreased risk. The dots are stacked vertically to show density. SHAP, Shapley Additive explanation; CR-POPF, clinically relevant postoperative pancreatic fistula; MPD, main pancreatic duct diameter; BMI, body mass index; ALT, alanine aminotransferase; PAR, platelet-to-albumin ratio.

**FIGURE 7:**
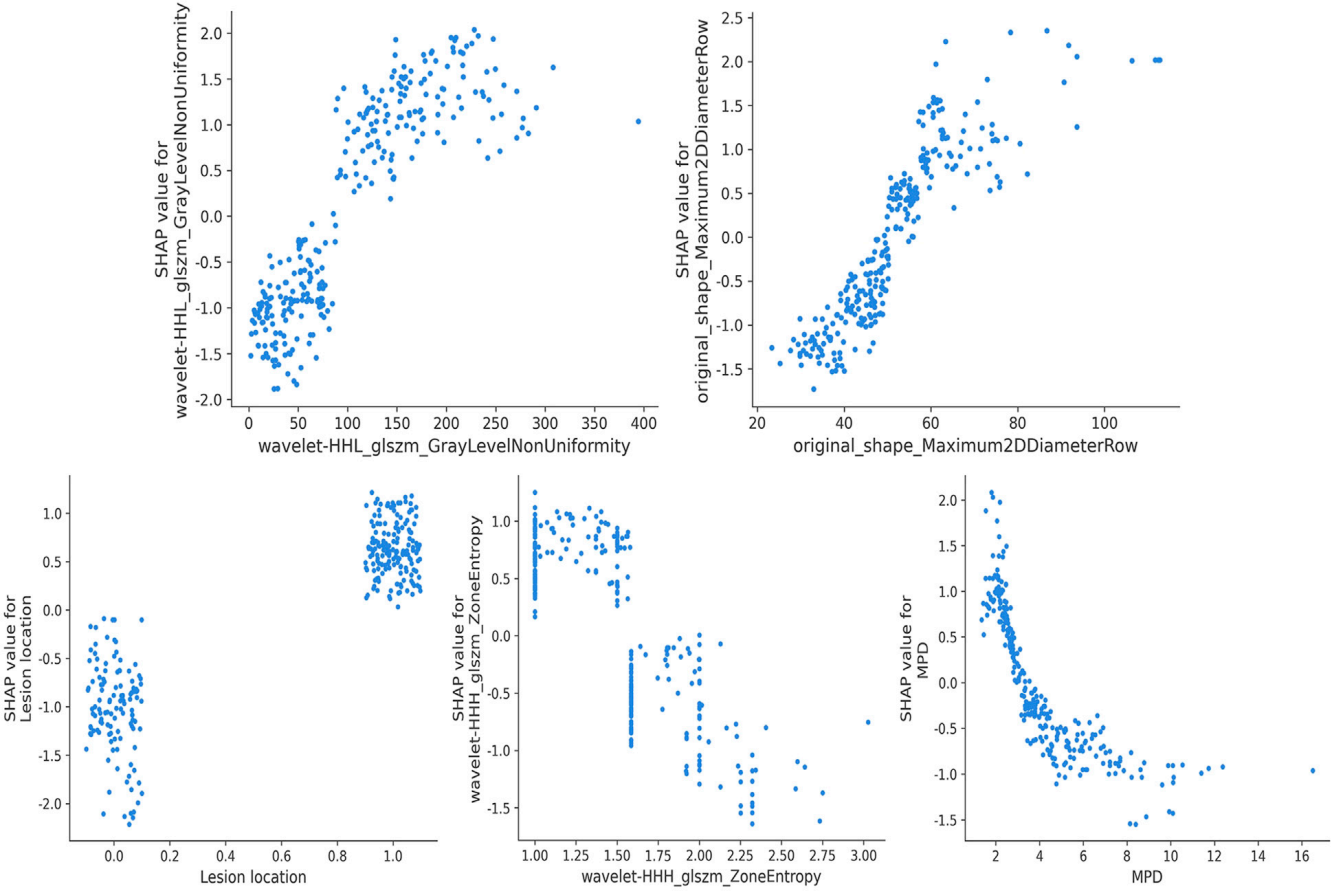
SHAP dependence plot. Each dependence plot shows how a single feature affects the output of the prediction model, and each dot represents a single patient. SHAP, Shapley Additive explanation; MPD, main pancreatic duct diameter.

**FIGURE 8:**
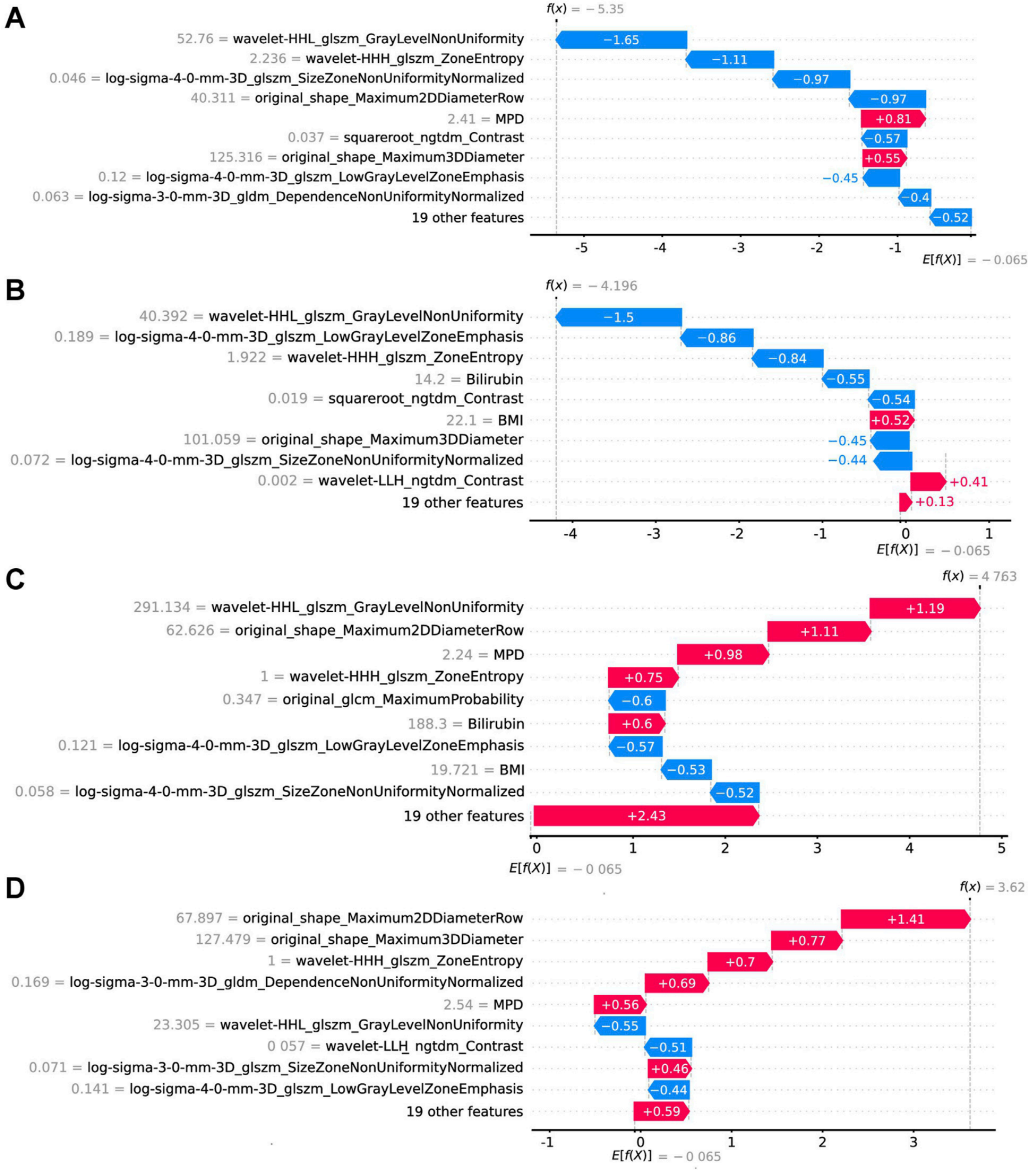
Local model explanation by the SHAP method. The SHAP waterfall plots illustrate how each feature contributes to individual predictions [**(A, B)** are CR-POPF negative cases; **(C, D)** are CR-POPF positive cases]. On a waterfall plot, the value at the bottom represents the expected value of the model output, and each row represents the contribution of each feature to the model output. A red arrow indicates an increased risk of CR-POPF, while a blue arrow indicates a decreased risk. The gray text before the feature names shows the value of each feature for the case. SHAP, Shapley Additive explanation; CR-POPF, clinically relevant postoperative pancreatic fistula; MPD, main pancreatic duct diameter; BMI, body mass index.

In the implementation of the XGBoost ensemble model, the SHAP method is utilized to elucidate the final model output through the computation of each variable’s contribution to the prediction. This interpretive strategy yields two categories of explanations: global explanations at the feature level and local explanations at the individual level. Global explanations elucidate the comprehensive behavior of the model and the significance of its features. This is illustrated in the SHAP bar chart and the SHAP summary plots (Figures 6A, C), where the influence of features on the model is assessed via mean SHAP values and presented in a descending sequence, thereby highlighting the top 20 variables that contribute most significantly to the model. The three variables with the highest contribution are wavelet-HHL_glszm_GrayLevelNonUniformity, original_shape_Maximum2DDiameterRow, and lesion location. The SHAP heatmap (Figure 6B) visually represents the direction and magnitude of the effect of each feature across all instances within the model. Additionally, SHAP dependence plots (Figure 7) facilitate comprehension of the manner in which a singular feature influences the output of the XGBoost predictive model. The y-axis denotes the SHAP value of the feature, in contrast to the x-axis, which signifies the value of the feature. The plot provides a visual representation of the fluctuating importance of the feature in relation to its value. A SHAP value exceeding zero corresponds to positive class predictions within the model, signifying an elevated risk of CR-POPF. Local explanations scrutinize the methods by which specific predictions for individual cases are formulated through the amalgamation of personalized input data. Figure 8 delineates instances of four standard positive and negative CR-POPF forecasts. The SHAP Waterfall plot elucidates the contributions of each attribute to the prediction outcome for a singular case. The baseline value symbolizes the model’s fundamental prediction probability, while each feature’s contribution value (also known as the SHAP value) signifies the direction and magnitude of that particular feature’s influence on the prediction. Positive values imply that the feature escalates the likelihood of predicting positive CR-POPF. The final prediction probability, denoted as f(x), is the cumulative sum of the baseline value and all feature contributions.

## 4 Discussion

### 4.1 Synergistic feature selection strategy

The integration of radiomics and clinical features through ML offers a transformative approach for preoperative prediction of CR-POPF. In this study, we extracted 1,719 radiomics features from preoperative portal venous phase CT images of 241 PD patients and combined them with clinical variables to develop a multimodal predictive model. The dual application of Lasso regression and RF algorithm for feature selection proved instrumental in balancing dimensionality reduction with biological relevance. Lasso’s regularization properties efficiently distilled 1,719 radiomics features to 20 non-redundant predictors, mitigating overfitting while preserving texture and shape parameters critical for quantifying pancreatic heterogeneity—a strategy validated in pancreatic cancer studies by Kim et al. (2019). Meanwhile, RF’s inherent ability to rank nonlinear interactions among clinical variables identified MPD, lesion location, PAR, and other important features as key contributors, reflecting anatomical risk and systemic inflammation, respectively (Huang et al., 2022; Tan et al., 2022). This hybrid approach harmonizes the strengths of both methods: Lasso’s sparsity induction for dimensionality reduction and RF’s robustness in handling multicollinearity, aligning with methodological frameworks advocating combined techniques for high-dimensional biomedical data (Azodi et al., 2020; Kumarasamy et al., 2021).

### 4.2 Performance comparison between unimodal models

The experimental results underscore the differential performance of ML predictors when utilizing single-modal versus multimodal features. Models trained solely on selected radiomics features achieved moderate predictive accuracy (AUC: 0.74–0.87), with texture parameters such as GLSZM and Gray Level Dependence Matrix (GLDM) emerging as pivotal predictors, consistent with studies emphasizing their utility in quantifying tissue heterogeneity and fibrosis—key determinants of pancreatic anastomotic integrity (Lubner et al., 2017; Chitalia and Kontos, 2019; Kim et al., 2019). For instance, Abunahel et al. linked GLSZM features to pancreatic stiffness, a surrogate for soft pancreatic texture widely associated with CR-POPF (Abunahel et al., 2022). Similarly, Capretti et al. reported comparable AUCs (0.75–0.81) using CT texture analysis, underscoring the reproducibility of radiomics in pancreatic risk stratification (Capretti et al., 2022). However, the inherent limitations of unimodal radiomics models—such as their inability to incorporate systemic physiological variables—highlight the necessity of integrating clinical data to enhance generalizability. Our clinical-only model, incorporating variables such as MPD diameter, lesion location, and PAR, achieved an AUC of 0.82–0.85. While this performance aligns with the predictive capacity of established risk scores like the FRS and updated alternative FRS (ua-FRS) (Mungroop et al., 2019; Mungroop et al., 2021), it demonstrates a moderate improvement over their external validation results (AUC: 0.74–0.82), highlighting the potential advantages of integrating modern ML frameworks with preoperative clinical indices. Notably, the significantly smaller MPD diameter in CR-POPF patients (2.77 vs 4.25 mm, P < 0.001) reflects multifactorial pathophysiology involving impaired drainage, reduced fibrosis-mediated anastomotic stability, and elevated duct-to-mucosa tension, synergistically increasing fistula risk (Casciani et al., 2021; Lee et al., 2023). Despite these strengths, clinical models struggle to capture subvisual parenchymal changes, such as microlobular fat infiltration or ductal microcalcifications, which radiomics excels in detecting (Chitalia and Kontos, 2019; Abunahel et al., 2022). This limitation highlights the necessity of integrating multimodal data to address the multifactorial nature of CR-POPF pathogenesis.

### 4.3 Superiority and interpretability of the combined model

The multimodal XGBoost model (AUC: 0.93) outperformed all unimodal approaches, underscoring the synergistic value of combining radiomics and clinical data. This aligns with emerging paradigms in precision oncology, where combined models consistently outperform unimodal approaches by encapsulating both macroscopic pathophysiology and microenvironmental heterogeneity (Shen et al., 2022; Verma et al., 2024). However, ML techniques are frequently characterized as “black boxes,” with limited studies dedicated to elucidating the sources of their predictions. This underscores an additional advantage of our study: following the training and evaluation of the model, we employed SHAP methods to interpret the “black box” nature of the ML model. By presenting the SHAP values, we elucidated the relationship between critical covariates and the estimated risk of CR-POPF: wavelet-HHL_glszm_GrayLevelNonUniformity (reflecting parenchymal disorganization) and MPD diameter jointly drove predictions, mirroring the interplay between ductal anatomy and tissue integrity. Such findings resonate with Lambin et al.’s assertion that radiomics bridges qualitative imaging and quantitative biology, thereby advancing clinical decision-making (Lambin et al., 2017).

### 4.4 Clinical implications for personalized prevention

Furthermore, case analysis elucidates the contributions of critical features within individual cases and computes the final Shapley values to derive the ultimate prediction probabilities, thereby facilitating personalized predictions. For patients at high risk of CR-POPF, preoperative preventive strategies, including nutritional support, optimization of diabetes and exocrine insufficiency, and respiratory training, may confer substantial benefits (Ausania et al., 2019; Bundred et al., 2020). Additionally, prophylactic medications, such as somatostatin analogs or hydrocortisone, have demonstrated efficacy in reducing complications associated with pancreatic surgery (Allen et al., 2014; Laaninen et al., 2016; Tarvainen et al., 2020). Risk assessment identifies patients best suited for interventions, cutting unnecessary medication costs. Evaluating the risk of CR-POPF also facilitates the management of drainage by enabling the early removal of drains in low-risk patients, consequently diminishing the risks of infection and erosion (Conlon et al., 2001; McMillan et al., 2017). Such personalized preventive measures are essential for mitigating the adverse effects associated with CR-POPF.

### 4.5 Limitations and future directions

Undeniably, our study has several limitations. First, the retrospective study design may introduce selection bias. Second, due to the model being derived from a single center, the sample size is relatively small, and external applicability needs further testing. Third, although our ML model can be used to assess the risk of CR-POPF in precision medicine, too many features limit its clinical application. Future studies should validate this framework prospectively and explore streamlined feature sets to facilitate real-world deployment.

## 5 Conclusion

This study presents a novel machine learning framework for preoperative prediction of CR-POPF by integrating CT radiomics and clinical features. The model leverages radiomic signatures, such as parenchymal heterogeneity, alongside clinical predictors, including MPD diameter and platelet-to-albumin ratio, achieving superior predictive performance with an AUC of 0.93. Enhanced interpretability is provided through SHAP, which identifies critical feature contributions, such as wavelet-HHL_glszm_GrayLevelNonUniformity, and enables patient-specific risk stratification. The framework offers significant clinical applicability, supporting perioperative interventions like prophylactic medication and optimized drain management to reduce morbidity. By combining quantitative imaging with actionable insights, this work advances precision surgery and highlights the transformative potential of explainable AI in pancreatic surgical oncology.

## Data Availability

The original contributions presented in the study are included in the article/Supplementary Material, further inquiries can be directed to the corresponding authors.
