# Supplementary figures and images for: Enhanced preoperative prediction of pancreatic fistula using radiomics and clinical features with SHAP visualization
Source: Front Bioeng Biotechnol. 2025 Apr 4;13:1510642. doi: 10.3389/fbioe.2025.1510642 (PMC12006764; doi:10.3389/fbioe.2025.1510642)

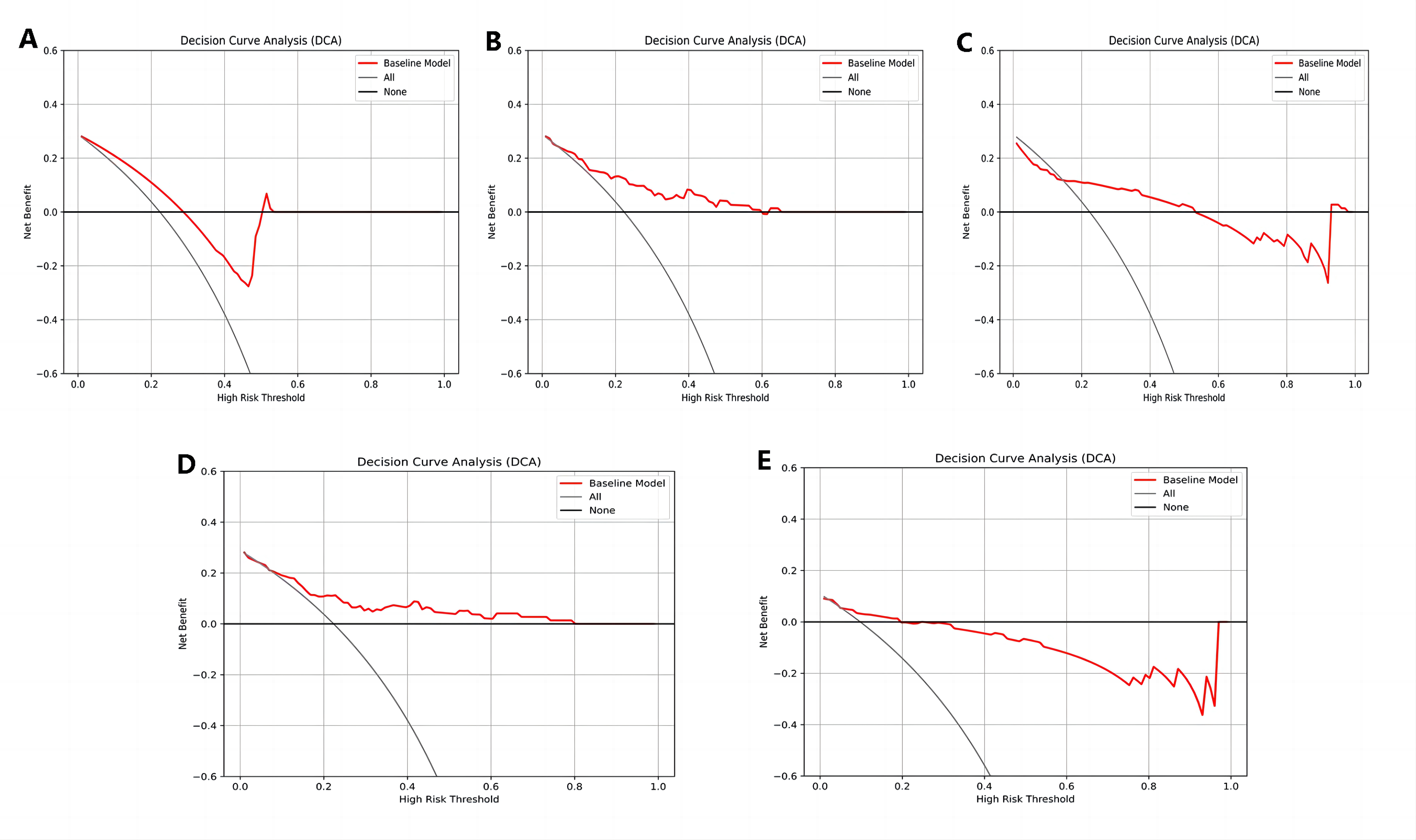

Supplement: Supplementary file 1 [file Image3.jpeg]

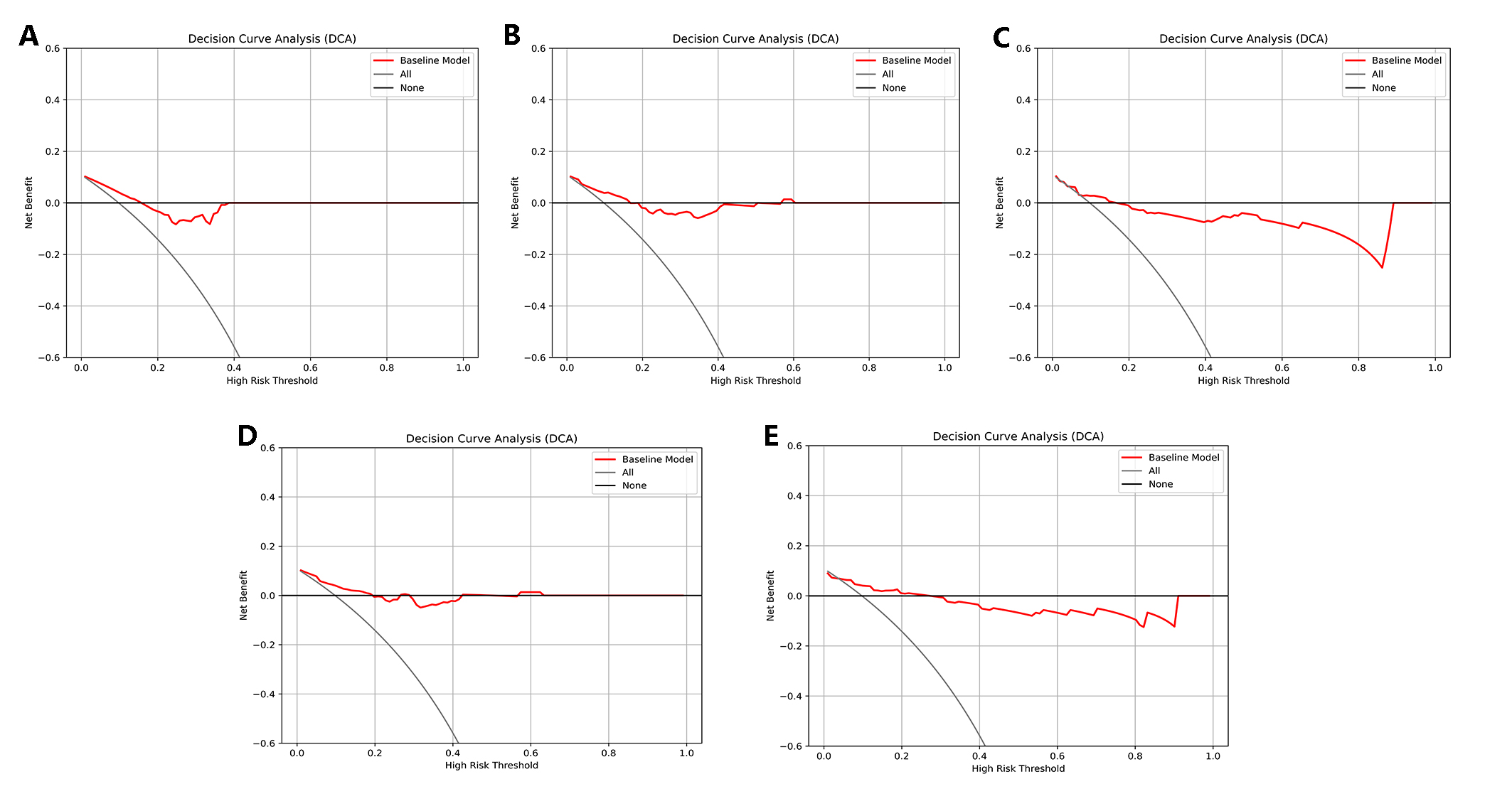

Supplement: Supplementary file 3 [file Image1.jpeg]

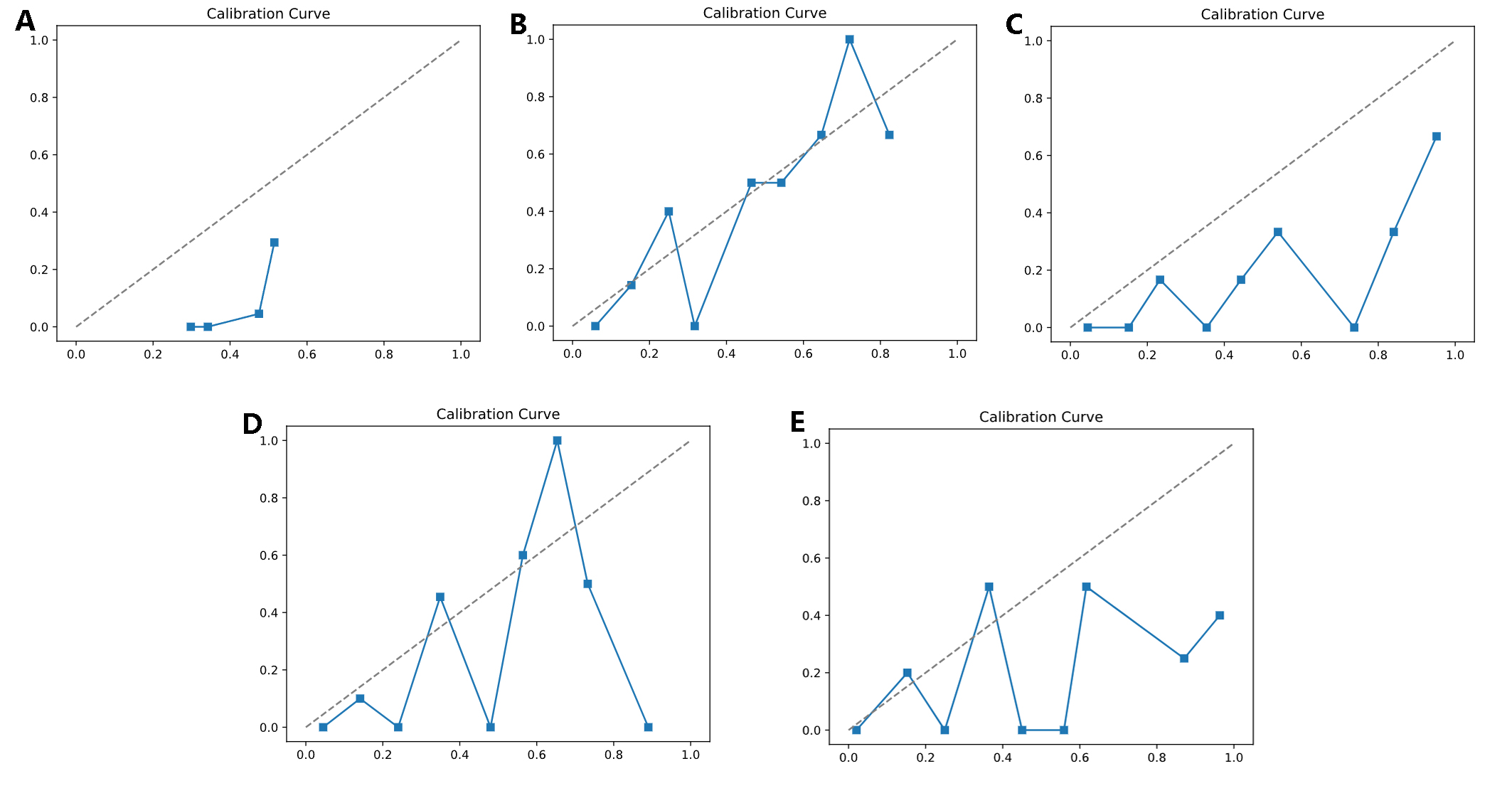

Supplement: Supplementary file 4 [file Image4.jpeg]

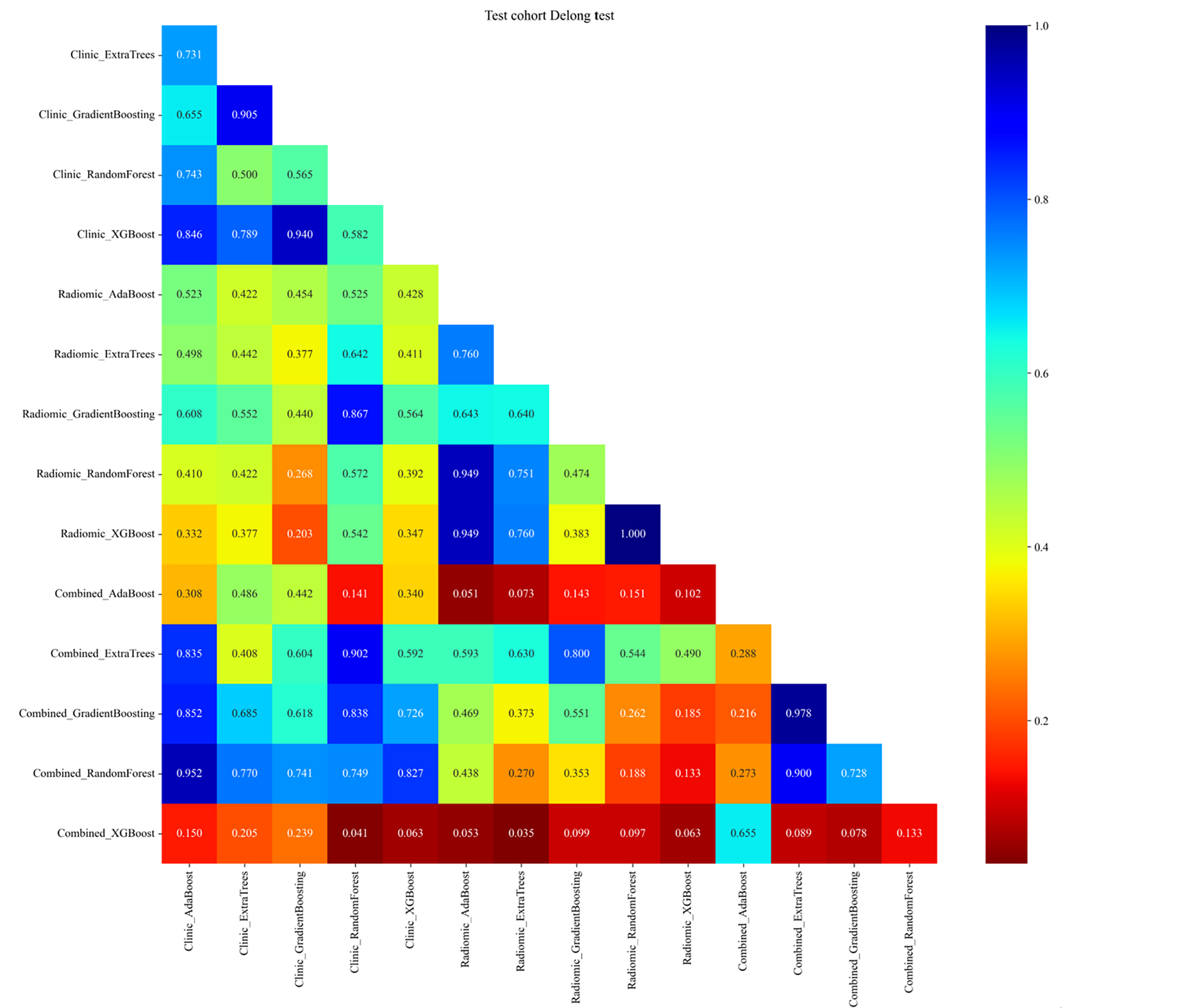

Supplement: Supplementary file 5 [file Image7.jpeg]

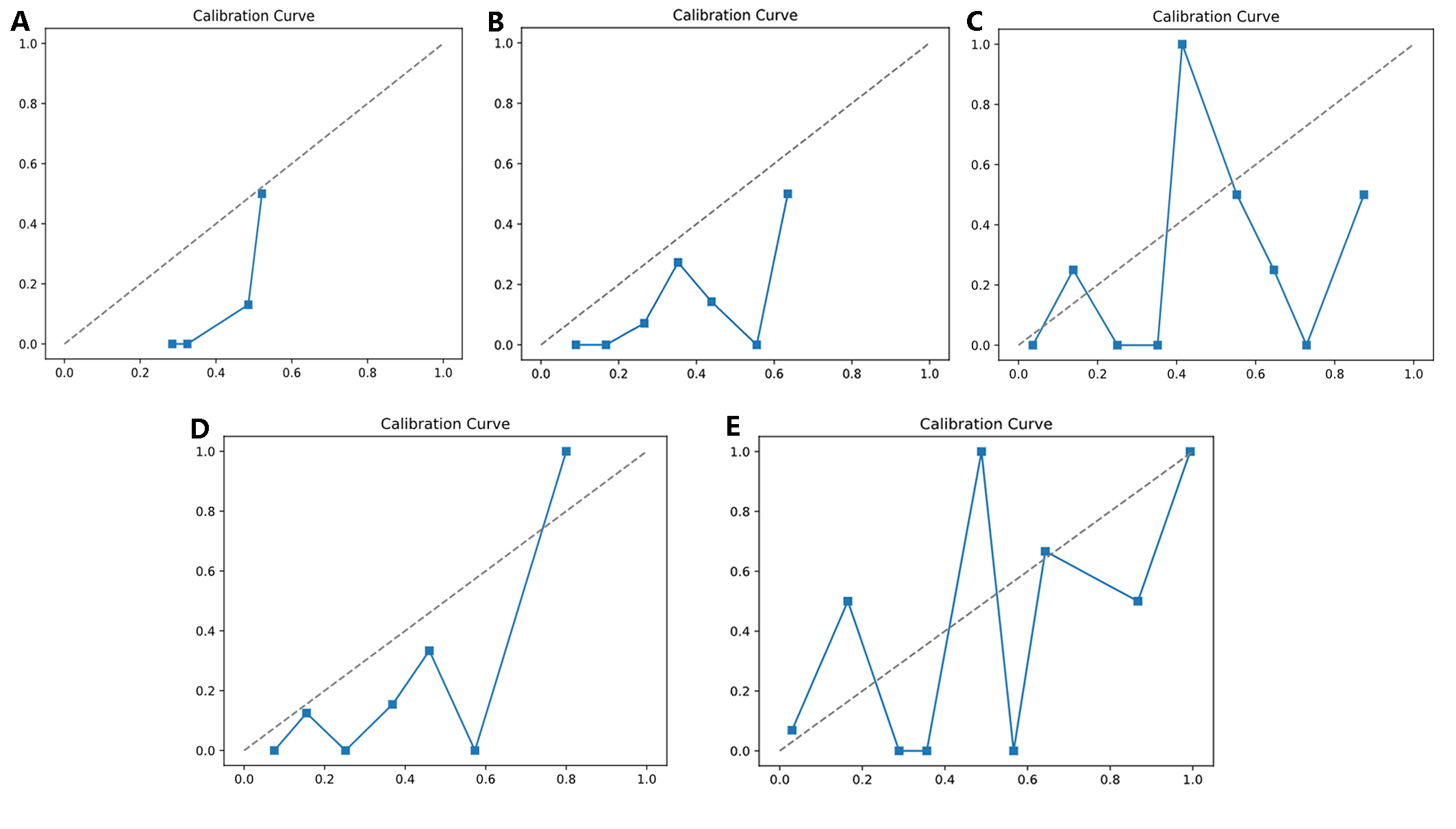

Supplement: Supplementary file 6 [file Image2.jpeg]

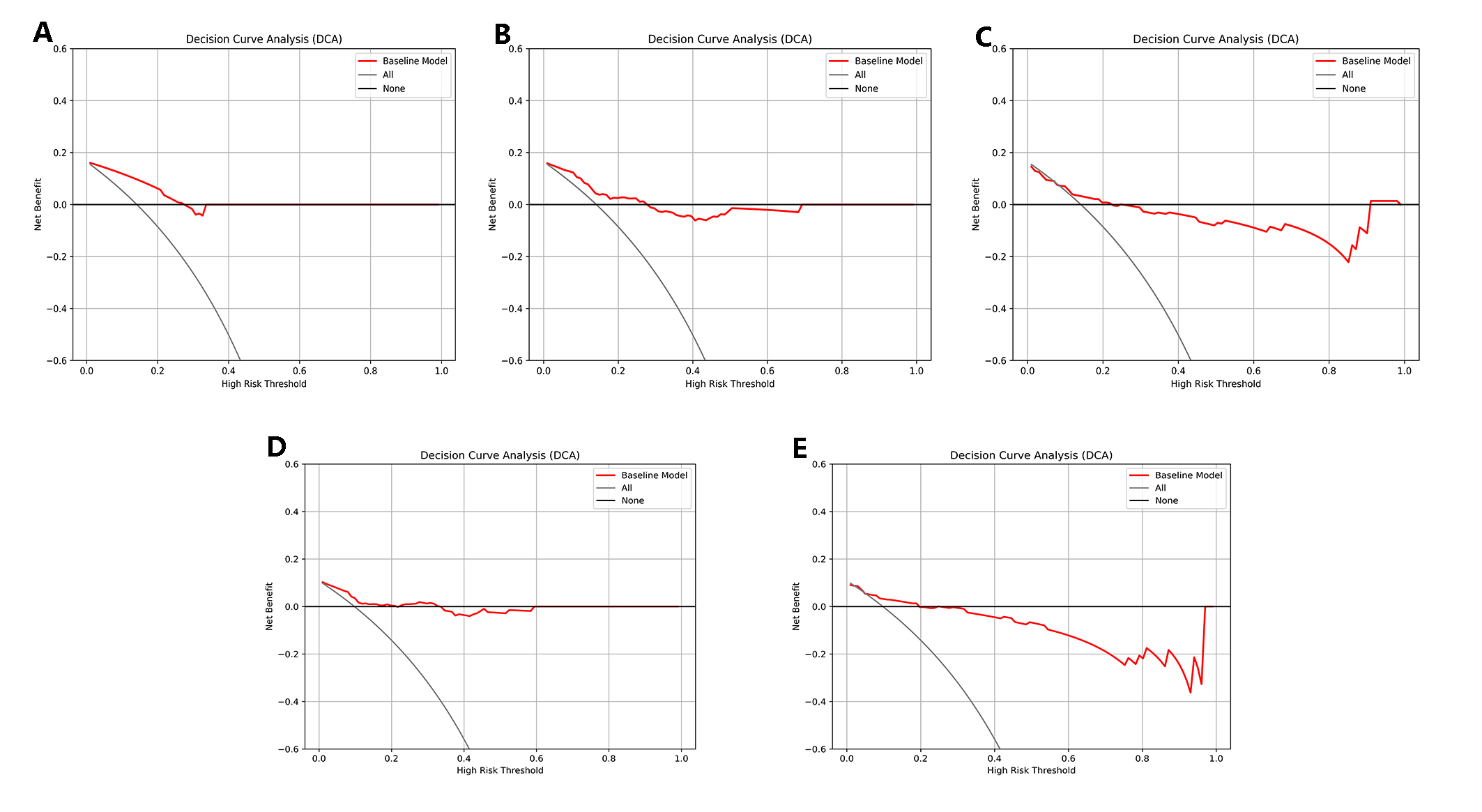

Supplement: Supplementary file 7 [file Image5.jpeg]

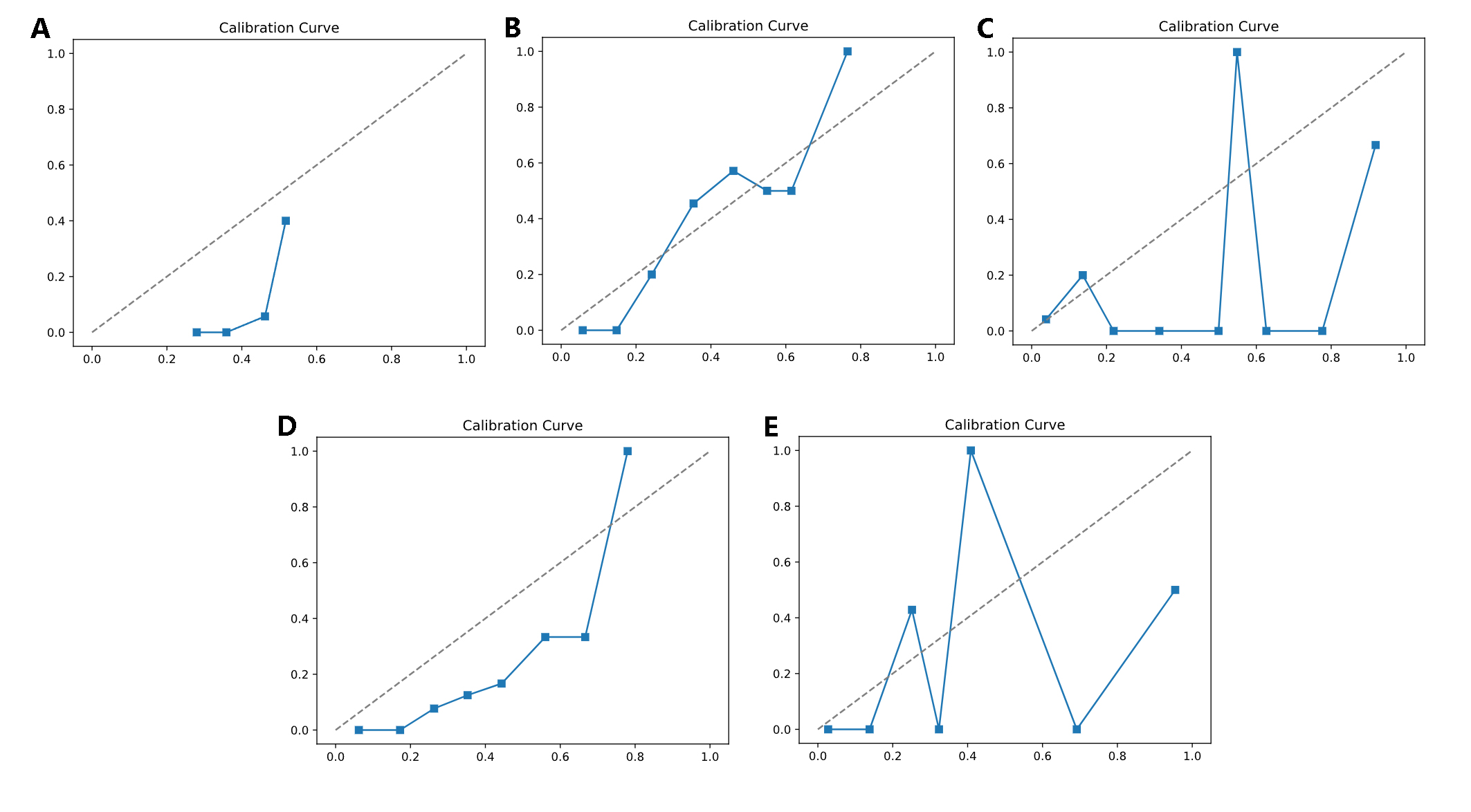

Supplement: Supplementary file 10 [file Image6.jpeg]
