# Supplementary material for: Enhanced preoperative prediction of pancreatic fistula using radiomics and clinical features with SHAP visualization
Source: Front Bioeng Biotechnol. 2025 Apr 4;13:1510642. doi: 10.3389/fbioe.2025.1510642 (PMC12006764; doi:10.3389/fbioe.2025.1510642)
Supplement: Supplementary file 2 [file DataSheet3.docx]

Supplementary Material 2:

Figure 1. The decision curves of the Radiomics-based prediction models for AdaBoost (A), Extra Trees (B), Gradient Boosting (C), Random Forest (D), and XGBoost (E) are shown.

Figure 2. The calibration curves of the Radiomics-based prediction models for AdaBoost (A), Extra Trees (B), Gradient Boosting (C), Random Forest (D), and XGBoost (E) are shown.

Figure 3. The decision curves of the Clinical Index-based prediction models for AdaBoost (A), Extra Trees (B), Gradient Boosting (C), Random Forest (D), and XGBoost (E) are shown.

Figure 4. The calibration curves of the Clinical Index-based prediction models for AdaBoost (A), Extra Trees (B), Gradient Boosting (C), Random Forest (D), and XGBoost (E) are shown.

Figure 5. The decision curves of the Radiomics-Clinical prediction models for AdaBoost (A), Extra Trees (B), Gradient Boosting (C), Random Forest (D), and XGBoost (E) are shown.

Figure 6. The calibration curves of the Radiomics-based prediction models for AdaBoost (A), Extra Trees (B), Gradient Boosting (C), Random Forest (D), and XGBoost (E) are shown.
