# Supplementary material for: Enhanced preoperative prediction of pancreatic fistula using radiomics and clinical features with SHAP visualization
Source: Front Bioeng Biotechnol. 2025 Apr 4;13:1510642. doi: 10.3389/fbioe.2025.1510642 (PMC12006764; doi:10.3389/fbioe.2025.1510642)

Supplementary Material 1:

Figure. Heatmap of pairwise AUC comparisons between different predictive models using DeLong’s test. The heatmap displays p-values for the AUC differences between various clinical, radiomic, and combined machine learning models. Significant comparisons (p < 0.05) are highlighted by red colors, indicating statistically significant differences in performance between models.


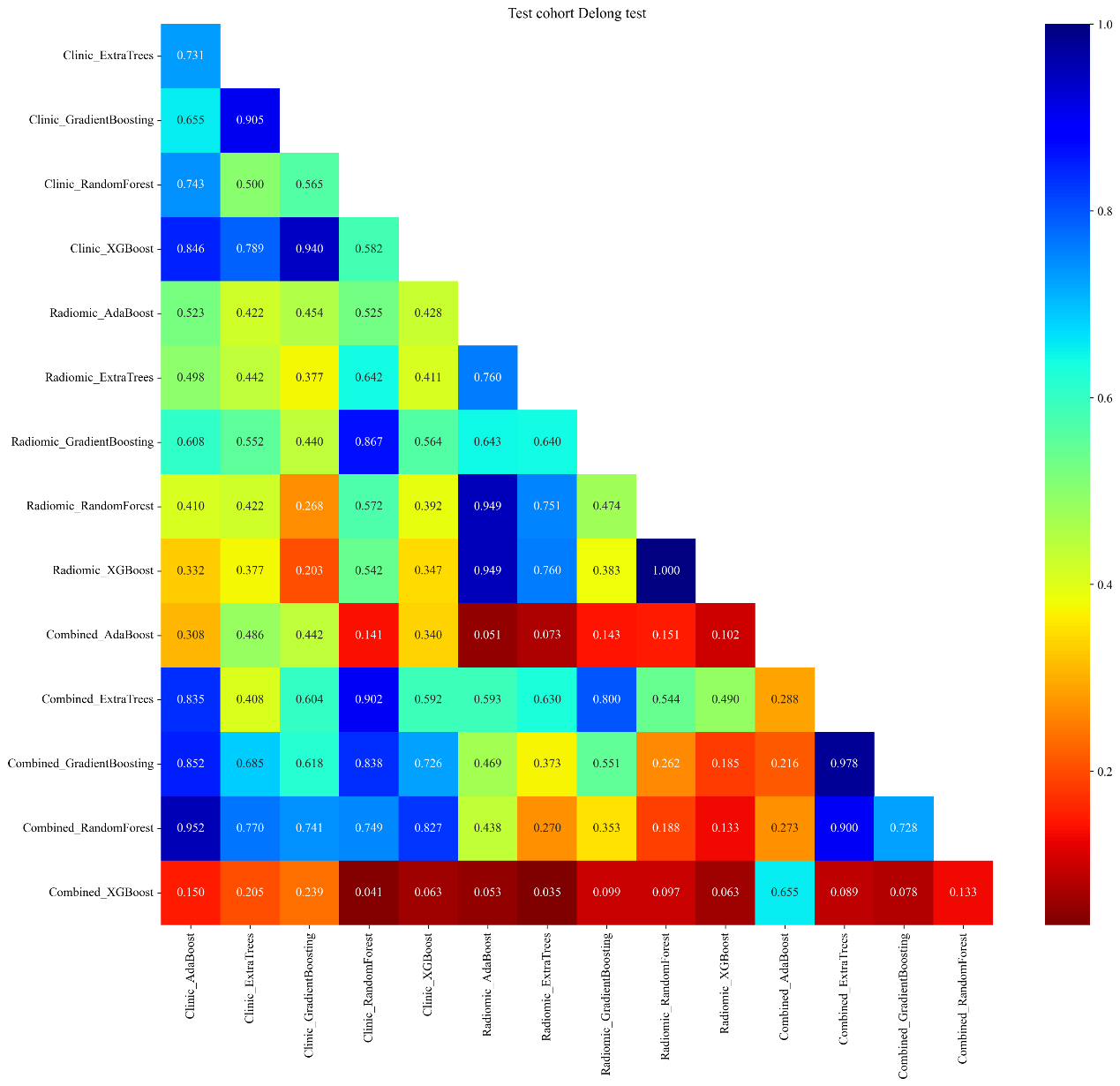

Supplement: Supplementary file 8 [file DataSheet2.docx]
